# Empathic disequilibrium in two different measures of empathy predicts autism traits in neurotypical population

**DOI:** 10.1186/s13229-020-00362-1

**Published:** 2020-07-13

**Authors:** Ido Shalev, Florina Uzefovsky

**Affiliations:** 1 Department of Psychology Ben Gurion University of the Negev, 84105 Beersheba, Israel; 2 Zlotowski Center for Neuroscience Ben Gurion University of the Negev, Beersheba, Israel

**Keywords:** Emotional empathy, Cognitive empathy, Autism, Broad autism phenotype

## Abstract

**Background:**

Features of autism spectrum conditions (ASC) are normally distributed within the population, giving rise to the notion of the autism spectrum. One of the hallmark features of ASC is difficulties in social communication, which relies heavily on our ability to empathize with others. Empathy comprises of both cognitive (CE) and emotional (EE) components that, together, allow us to understand another’s emotions and be affected by them appropriately, while maintaining a self-other distinction. Although CE and EE depend on distinct neural and developmental trajectories, it was suggested that the two empathic capacities can influence, balance, and regulate each other. Previous findings regarding the role of emotional and cognitive empathy in ASC have been mixed. Therefore, our study aimed to investigate whether the intra-personal empathy imbalance between the cognitive and emotional components, a measure we termed empathic disequilibrium (ED), can be associated with autism traits at the neurotypical range.

**Methods:**

Participants were 671 young-adults at the neurotypical range who self-reported their empathy, assessed using two highly validated questionnaires—the Interpersonal Reactivity Index and the Empathy Quotient, autism traits using the Autism-Spectrum Quotient, and the related traits, alexithymia, and systemizing.

**Results:**

Controlling for the total empathy score, greater ED was found to be positively correlated with autism traits. Specifically, autism traits were found to be elevated in groups of individuals with relatively higher EE than CE, underscoring their imbalance.

**Conclusions:**

Our study offers a novel perspective on the understanding of the social difficulties associated with autism tendencies in the general population and has potentially important clinical implications for understanding of ASC. We also propose a novel characterization of autism tendencies based on the imbalance between EE and CE, which we term ED, as opposed to examining EE and CE separately.

## Background

Autism spectrum conditions (ASC) are a behaviorally diagnosed set of neurodevelopmental conditions characterized by difficulties in social interaction and communication, accompanied by repetitive and restrictive behaviors with onset during early development [1]. Individuals with ASC display considerable variability in many features including cognitive, emotional, biological, and developmental aspects [2–4]. This phenotypic heterogeneity suggests that ASC is indeed heterogeneous, representing an aggregation of multiple conditions [5]. Studies consistently show that milder, yet similar, characteristics of the defining features of ASC are continuously distributed across the general population, representing both familial and genetic liability to ASC [6, 7]. Accordingly, individuals with a clinical diagnosis of ASC are viewed as the extreme end of a continuum of autism traits, which at some point cross the line into the clinical phenotype [8]. Moreover, similar to ASC phenotypes, this subclinical population also presents significant heterogeneity [9, 10].

Therefore, studying the autism spectrum in the general population is informative to clarifying the multiple characteristics in individuals who are genetically and epigenetically predisposed to ASC, thus elucidating the nature of ASC and its variability [11]. The aim of the current study was to focus on the origins of the social difficulties related to ASC by examining whether disequilibrium in emotional versus cognitive empathy contributes to autism tendencies in the general population.

Although tremendous variability is evidenced in ASC phenotypes, difficulties in social communication are indisputably fundamental to its definition [12]. Human ability for social communication relies heavily on our ability to empathize with others [13]. Consistently, many studies suggest that deficits in empathy are prevalent in autism [14–17]. Therefore, studying the relationship between empathy and autism tendencies can inform our understanding of ASC.

### Emotional and cognitive empathy

Empathy, defined as the ability to understand another’s emotions and be affected by them appropriately, while maintaining a self-other distinction, includes both emotional and cognitive components [18]. Emotional empathy (EE) is the ability to respond to another’s mental states with an appropriate emotion, while cognitive empathy (CE) is the ability to recognize what another person is feeling [14]. CE is closely related to the affective aspects of Theory of Mind, which is defined as the ability to make inferences regarding other’s emotions [19], whereas EE includes an emotional response to other’s internal states [20]. Oftentimes, the measurement of EE focuses on the emotional response to another’s distress, which can take two forms. One form is that of feeling of compassion or concern towards the other, termed sympathy or empathic concern. Another form is the tendency to experience distress or discomfort in response to distress in others, termed personal distress [21].

CE and EE have different developmental and neural trajectories. For instance, CE continuously increases throughout early childhood and adolescence, while EE appears very early on and remains relatively stable throughout the lifespan [22–25]. Furthermore, neuro-imaging studies suggest that CE tasks activate brain regions such as ventromedial prefrontal cortex and the temporo-parietal junction, while EE tasks relate to the amygdala and cingulate cortex structures [19, 26–28]. The distinction between the two empathy components has been supported by genetic studies as well [29–32].

Although CE and EE depend on distinct neural networks, overlapping neural response between the two empathic capacities was found in brain regions such as the anterior insula [33, 34]. Accordingly, it was suggested that EE and CE can influence, balance, and regulate each other while simultaneously retaining a significant degree of independence and that both functions are jointly required in complex social situations [35–38]. This has been shown in a meta-analysis study of empathy for pain, in which brain activation was compared between two types of empathy tasks [28]. In a picture-based task, participants were presented with visual depictions of someone in a painful situation, while in the cue-based task, participants were presented with only a cue/hint that someone else is receiving painful stimulation. In addition to a common activation in the two paradigms, CE and EE brain regions were co-activated in the cue-based task. This suggests that CE-related brain regions interact with EE-related brain regions in complex and unclear or ambiguous social situations, in which additional processing is needed to jointly engage EE and CE with the feelings of the other. As complex and relatively ambiguous social situations are constantly encountered in everyday life, these studies suggest that maintaining a balance between EE and CE is crucial for an adaptive and appropriate social response, leading to effective social communication.

Relatedly, deficits in CE and EE were found to be dissociable in a wide array of psychiatric conditions including schizophrenia [39], anti-social personality disorder [16], obsessive-compulsive disorder [40], and bipolar disorder [41].

### Emotional and cognitive empathy in ASC

The dissociation between CE and EE was also suggested to be a hallmark of individuals with ASC [14]. The empathy imbalance hypothesis (EIH) proposes that individuals with ASC show impaired CE, while maintaining high EE functioning and that this imbalance contributes to some autism symptoms [36]. In line with this hypothesis, many empirical findings based on self-report [42, 43] and neurobehavioral [15, 44, 45] measures of empathy show impaired CE in individuals with ASC, while EE is either exaggerated or remains intact.

While these studies converge on a specific pattern of empathy imbalance in individuals with ASC, others show mixed and inconsistent results [17, 46, 47]. For instance, one study [46] found that young children with autism displayed EE less frequently compared to children at the neurotypical range. This reduced responsiveness could not be explained by a failure to look at the experimenters’ emotional displays. In another study [4], the Reading the Mind in the Eyes Test (RMET), a task measuring CE by asking participants to recognize others’ mental states from the eyes region of the face [48, 49], was used to classify individuals with ASC into five separate subgroups, two of which did not differ in RMET score from individuals at the neurotypical range. The researchers suggested the notion that there are ASC subgroups which do not show lower CE.

One explanation for these findings might be that CE and EE by themselves are not sensitive enough to characterize ASC, and there is a need to consider the intra-personal variability between EE and CE, while previous studies examined EE and CE separately. For example, we suggest that individuals showing average CE, are also characterized by even higher EE and it is the imbalance between the traits which is at the root of the social difficulties displayed in autism. Thus, we suggest there is a need to jointly examine the balance between CE and EE, and not, as carried out in most previous investigations, only each of these components individually.

To our knowledge, currently, only one empirical study quantified the intra-personal imbalance between CE and EE directly [50]. In this study, the Interpersonal Reactivity Index (IRI) [21], a commonly used self-report measure of empathy, was used to derive a new measure investigating the intra-personal empathy of each individual, termed “relative empathic ability” (REA). The researchers showed differences in functional connectivity between individuals with EE-dominance, showing stronger connectivity among social-emotional regions, and individuals with CE-dominance, showing stronger social-cognitive processing and interoceptive network connectivity. REA was also associated with some symptoms of psychopathology, that could not be otherwise explained by CE and EE separately. Although this study found no association between REA and autism traits in participants at the neurotypical range, their analysis was based on only 18 participants, a very small sample size likely lacking power to detect the sought after effect, suggesting that further investigations, with larger sample size, are needed.

### The current study

Our study aimed to investigate whether intra-personal empathy imbalance can be associated with autism traits in the general population. Based on Cox et al.’s [50] findings regarding the neurobiological and behavioral implication of REA, and the empathic imbalance hypothesis [36], we preferred to use the term “empathic disequilibrium” (ED) to define this imbalance, thus capturing its possible clinical implications. For our purpose, we created an ED measure, derived separately from two highly validated self-report questionnaires of empathy, the IRI [21] and the Empathy Quotient (EQ) [14]. We used both measures separately to make sure that findings are not measure-dependent. We hypothesized that ED will be positively correlated with autism traits in the general population. We also explored the differences in autism traits between the two ED groups of individuals (EE-dominant and CE-dominant). To better define each group, we further investigated other autism-related traits that were previously proposed to characterize ASC and its subgroups including alexithymia, a subclinical condition characterized by difficulties in identifying and describing one’s own emotional state [51] and systemizing, the drive to analyze, or construct systems [52, 53].

## Methods

### Participants

A total of 671 college students (56% females; mean age 24.5 **±** 2.5) were recruited by word of mouth and advertisements on Israeli institution campuses. All of the participants filled out the EQ, and 629 of those completed the full battery of questionnaires, as listed below. One participant was removed from the IRI analyses due to unusually low EE and CE scores on the questionnaire (< − 4 standard deviations), leaving 628 participants in the IRI analyses. This number of participants should provide sufficient power to detect effects of small size (*f*^2^ > 0.02). Participants were of Jewish descent, with no self-reported history of psychiatric disorders, chronic illness, or drug taking. All participants were paid volunteers. This sample was previously analyzed and described by Uzefovsky et al. [32]. As expected of the general population, all measurements including Autism-Spectrum Quotient, Toronto Alexithymia Scale, Systemizing Quotient, EQ, and IRI in this sample (see Table 1 for means) fell within the average range previously reported in neurotypical population [21, 53–55] with only five participants who scored above, or equal to, the Autism-Spectrum Quotient clinical cut-off score of 32 [56].

**Table 1:** Descriptive statistics—measures of autism-related traits and empathy

		Subscale	Mean	Sd
Measure	**AQ**		19.19	4.44
		Social skill	3.41	1.4
		Attention switching	4.04	2.11
		Attention to detail	5.47	1.96
		Communication	3.2	1.45
		Imagination	3.07	1.54
	**TAS-20**		41.67	11.91
		Difficulty describing feelings	11.16	4.52
		Difficulty identifying feelings	14.02	5.52
		Externally-oriented thinking	16.48	4.79
	**SQ**		27.9	11.05
Empathy measure	**IRI**		94.46	11.47
		Perspective taking	25.13	4.21
		Fantasy	23.91	5.06
		Empathic concern	25.58	4.17
		Personal distress	19.84	4.03
	**EQ**		43.07	10.41
		Cognitive empathy	11.8	4.06
		Emotional empathy	12.59	4.53

Descriptive statistics of autism-related deficits, empathy measures, and their subscales. *AQ* Autism-Spectrum Quotient, *TAS-20* Toronto Alexithymia Scale, *SQ* Systemizing Quotient, *IRI* Interpersonal Reactivity Scale, *EQ* Empathy Quotient

### Measures

Participants came to the lab, where informed consent was obtained. Participants were then given access to an online, password-protected platform. There, participants filled out a demographic questionnaire and completed a battery of questionnaires measuring empathy and autism-related traits.

### Empathy measures

Empathy was measured using two different highly validated empathy questionnaires. These measures were analyzed according to validated emotional and cognitive empathy subscales.

#### Interpersonal reactivity index (IRI) [21]

The questionnaire consists of 28 items on a 5-point scale, which can be divided into four validated subscales, each made up of seven items. Two of the subscales measure CE (perspective taking (PT) and fantasizing (F)) and two subscales measure EE (empathic concern (EC) and personal distress (PD)).

#### Empathy quotient (EQ) [14]

The questionnaire consists of 60 items (40 empathy items and 20 filler items) on a 4-point scale. On each empathy item, a person can score 2, 1, or 0. The EQ consists of three validated factors: “cognitive empathy” (11 items), “emotional reactivity” (11 items), and “social skills” (6 items) [57, 58]. In the current study, we focused on the first two subscales and did not include “social skills” as it does not directly relate to EE or CE. The “emotional reactivity” scale was used as a measure of EE. An example of an item is “I tend to get emotionally involved with a friend’s problems”. The authors define emotional reactivity as the tendency to have an emotional reaction in response to others’ mental states, which is similar to the definition of EE. Although “emotional reactivity” does not include aspects of personal distress, as does the EE scale of the IRI, it does capture the sharing in other’s emotional experience. Thus, we treated this factor as a measure of EE, similarly to other numerous studies (e.g., [59–62]).

### Autism-related measures

#### Autism-Spectrum quotient (AQ) [56]

This questionnaire consists of 50-items measuring autism traits in the general population. All items in this measure are scored on a four-point rating scale, with higher total score indicating higher autism traits. The AQ can also be divided according to five domains: “social skill,” “attention switching,” “attention to detail,” “communication,” and “imagination.”

#### Toronto alexithymia scale (TAS-20) [63]

This is a 20-item measure designed to assess alexithymia, which is defined as a difficulty in describing and identifying one’s emotional state. A higher score on this questionnaire indicates higher alexithymia. TAS-20 has three subscales: “difficulty describing feelings,” “difficulty identifying feelings,” and “externally-oriented thinking” (made up of 5, 7, and 8 items, respectively).

#### Systemizing quotient (SQ) [53]

This is a 60-item (40 systemizing items and 20 filler items) questionnaire with a 0–2 rating scale aimed to assess systemizing disposition, which is the drive to analyze or construct systems.

Descriptive statistics of each measure used in this study are shown in Table 1. Raw Pearson’s correlations matrix of all the measures is shown in Table 2.

**Table 2:** Raw correlation matrix

	EQ	IRI	AQ	TAS-20	SQ
EQ	—				
IRI	0.47***	—			
AQ	− 0.28***	0.05	—		
TAS-20	− 0.49***	− 0.17***	0.3***	—	
SQ	0.05	− 0.13**	0.01	− 0.14***	—

Raw Pearson’s correlation matrix between each of the measures. *EQ* Empathy Quotient, *IRI* Interpersonal Reactivity Scale, *AQ* Autism-Spectrum Quotient, *TAS-20* Toronto Alexithymia Scale, *SQ* Systemizing Quotient

*p < 0.05, **p < 0.005, ***p < 0.0005

#### Empathic disequilibrium calculation

Using the CE and EE scores derived from each empathy questionnaire (IRI and EQ) separately, we created two indices to represent and quantify ED.

1. ED was calculated as (standardized CE score − standardized EE score), thus representing individuals’ relative differences (in standard deviation) between CE and EE. We used standardized scores as it provides a meaningful scoring system (a score of 1 represents one standard deviation difference between EE and CE) and allows to avoid biases that might affect the comparability between the two traits (e.g., subjects may be prone to expectancy bias in only one scale). In this measure, a positive score indicates *CE-dominance*, while a negative score indicates *EE-dominance*.
2. ED-magnitude is the ED score in absolute value, indicating the level of disparity between emotional and cognitive empathy. Thus, a high ED-magnitude score indicates an imbalance between cognitive and emotional empathy, while a low ED-magnitude score confers a balance between the two traits.

### Statistical analysis

Two types of analyses were preformed, for each of the empathy measures, to answer our main questions. (1) To examine whether ED-magnitude uniquely contributes to the prediction of autism traits, we conducted multiple regression analyses with ED-magnitude derived from IRI and EQ separately. In each analysis, we predicted AQ score using empathy score (from IRI or EQ) and its derivative ED-magnitude score. We controlled for sex and age in all analyses. To make sure ED-magnitude uniquely contributes to the prediction of autism traits, similar analyses were conducted with EE and CE instead of the general empathy score.

**Fig. 1:**
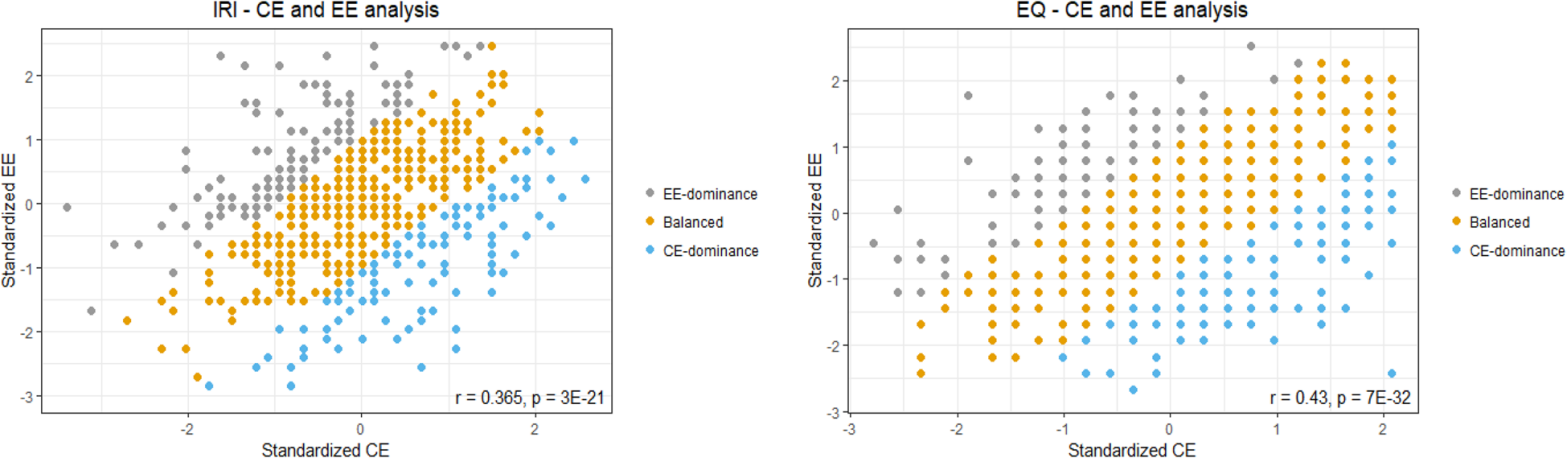
Correlation between CE and EE. Distribution of CE and EE scores derived from IRI (left panel) and EQ (right panel) per participant. Empathic (dis)equilibrium groups are represented using color, showing EE-dominance in grey, balanced empathy in orange, and CE-dominance in blue. CE, cognitive empathy; EE, emotional empathy; IRI, Interpersonal Reactivity Index; EQ, Empathy Quotient

(2) To examine the differences between CE-dominant versus EE-dominant ED scores, we grouped the participants according to their ED scores to CE-dominant (**≥** 1 SD), EE-dominant (≤ −1 SD) and balanced empathy (between − 1 and 1 SD) groups. Characteristics of participants in each group are shown in Table 3. A plot of the correlation between CE and EE scores, (*r* = 0.43, *p* = 7 × 10^−32^ in EQ; *r* = 0.365, *p* = 3 × 10^−21^ in IRI) grouped by ED dominance is shown in Fig. 1.

**Table 3:** Empathy group characteristics

IRI							
	**EE-dominance (*N*= 107)**		**Balanced (*N*= 409)**		**CE-dominance (*N*= 112)**		**Significance testing**
	**Mean**	**SD**	**Mean**	**SD**	**Mean**	**SD**	
IRI score	93.69	12.28	94.63	11.1	94.56	12.09	*F* = 0.29, *p* = 0.75
Age	24.2	2.74	24.5	2.4	24.68	2.71	*F* = 0.86, *p* = 0.43
Sex	*m* = 37, *f* = 69		*m* = 176, *f* = 233		*m* = 65, *f* = 47*		
	chi-square = 3.56, df = 1, *p* value = 0.059		chi-square = 0.16, df = 1, *p* value = 0.69		chi-square = 8.95, df = 1, *p* value = 0.003		
EQ							
	**EE-dominance (*N*= 110)**		**Balanced (*N*= 442)**		**CE-dominance (*N*= 119)**		**Significance testing**
	**Mean**	**SD**	**Mean**	**SD**	**Mean**	**SD**	
EQ score	42.59	9.73	43.72	10.61	41.09	10.1	*F* = 3.14, *p* = 0.044*
Age	24.25	2	24.55	2.67	24.44	2.36	*F* = 0.55, *p* = 0.57
SEX	*m* = 33, *f* = 76*		*m* = 182, *f* = 250		*m* = 74, *f* = 39*		
	chi-square = 8.33, df = 1, *p* value = 0.004		chi-square = 0.61, df = 1, *p* value = 0.43		chi-square = 21.17, df = 1, *p* value < 0.0001		

Characteristics of each empathy group were calculated using participants’ ED scores based on Interpersonal Reactivity Index (IRI; top table) and Empathy Quotient (EQ; bottom table). Number (*N*) of participants in each group is depicted in parenthesis. For each group, mean and standard deviation (SD) of total empathy score, age, and sex is depicted, as well as one-way ANOVA *p* value examining the differences in empathy score and age between the three groups. Chi-square test for goodness of fit was used to examine sex differences in each group. *p < 0.05.

Differences between the three groups (EE-dominance/CE-dominance/balanced empathy) in autism-related traits, measured by AQ, TAS-20, and SQ, were separately examined using a one-way ANOVA. Sex was used as a covariate. We assigned a strict Bonferroni-corrected *p* value of (0.05/3 tests = 0.017) to account for multiple testing. The analyses of the AQ and TAS-20 subscales can be found in Additional File 1. We further investigated the reliability of ED, by analyzing ED scores calculated using two additional methods. The first such ED was calculated based on a mean of the relevant subscales originating from the IRI and EQ. The second, based on participants whose group assignment to the ED groups was identical based on both measures. Details regarding these analyses and their outcomes appear in Additional File 1. All statistical analyses mentioned above were carried out using R v3.6.1 using the stats [64] and emmeans [65] packages.

## Results

### IRI-derived ED analyses

Applying multiple regression analysis with age and sex as covariates, ED-magnitude was found to be positively correlated with AQ score (*β* = 0.124, *p* = 0.004), while the total IRI score failed to predict AQ (*β* = 0.05, *p* = 0.24). In this analysis, AQ score was neither explained by age (*β* = 0.04, *p* = 0.32) nor sex (*β* = − 0.01, *p* = 0.8). Findings were similar when controlling for EE and CE (instead of a total empathy score); a positive correlation was observed between ED-magnitude and AQ score (*β* = 0.14, *p* = 0.001). Neither age (*β* = 0.04, *p* = 0.32) nor sex (*β* = − 0.01, *p* = 0.81) predicted AQ score. AQ score was negatively correlated with CE (*β* = − 0.21, *p* = 7 × 10^−6^), but no correlation was found between AQ score and EE (*β* = 0.06, *p* = 0.16).

We next grouped participants based on their ED scores. As described in Table 3, participants in each group did not differ in age (*F* = 0.86, *p* = 0.43, *ƞ*_*p*_^2^ = 0.003) and total IRI score (*F* = 0.29, *p* = 0.75, *ƞ*_*p*_^2^ < 0.001). Relative to the whole sample, sex differed in the CE-dominant ED group (χ^2^(1, *N* = 112) = 8.95, *p* = 0.003) so that this group had more males than females (58%).

Results of the one-way ANOVA examinations of autism traits between the three groups are described in Table 4 (AQ and TAS-20 subscales results are described in Supplementary Table 1 and Supplementary Table 2 in Additional File 1, respectively). These analyses revealed significant differences between the three groups in AQ, TAS-20, and SQ scores.

**Table 4:** IRI-derived ED analysis of empathy groups

	EE-dominance (***N*** = 107)		Balanced (***N*** = 409)		CE-dominance (***N*** = 112)		***p*** value	***F***	***η***_***p***_^**2**^
	Mean	SD	Mean	SD	Mean	SD			
AQ***	21.18	5.07	18.8	4.25	18.62	4.05	3 × 10^−6^	13.06	0.04
TAS-20***	45.76	12.19	41.8	11.9	37.33	10.2	5 × 10^−8^	17.26	0.053
SQ***	24.52	10.36	27.36	10.71	33.05	11.3	2 × 10^−6^	13.41	0.04

One-way ANOVA analyses results of the differences in AQ, TAS-20, and SQ scores between ED groups derived from IRI. *IRI* Interpersonal Reactivity Index, *AQ* Autism-Spectrum Quotient, *TAS-20* Toronto Alexithymia Scale, *SQ* Systemizing Quotient

*p < 0.017, **p < 0.0017, ***p < 0.00017

**Fig. 2:**
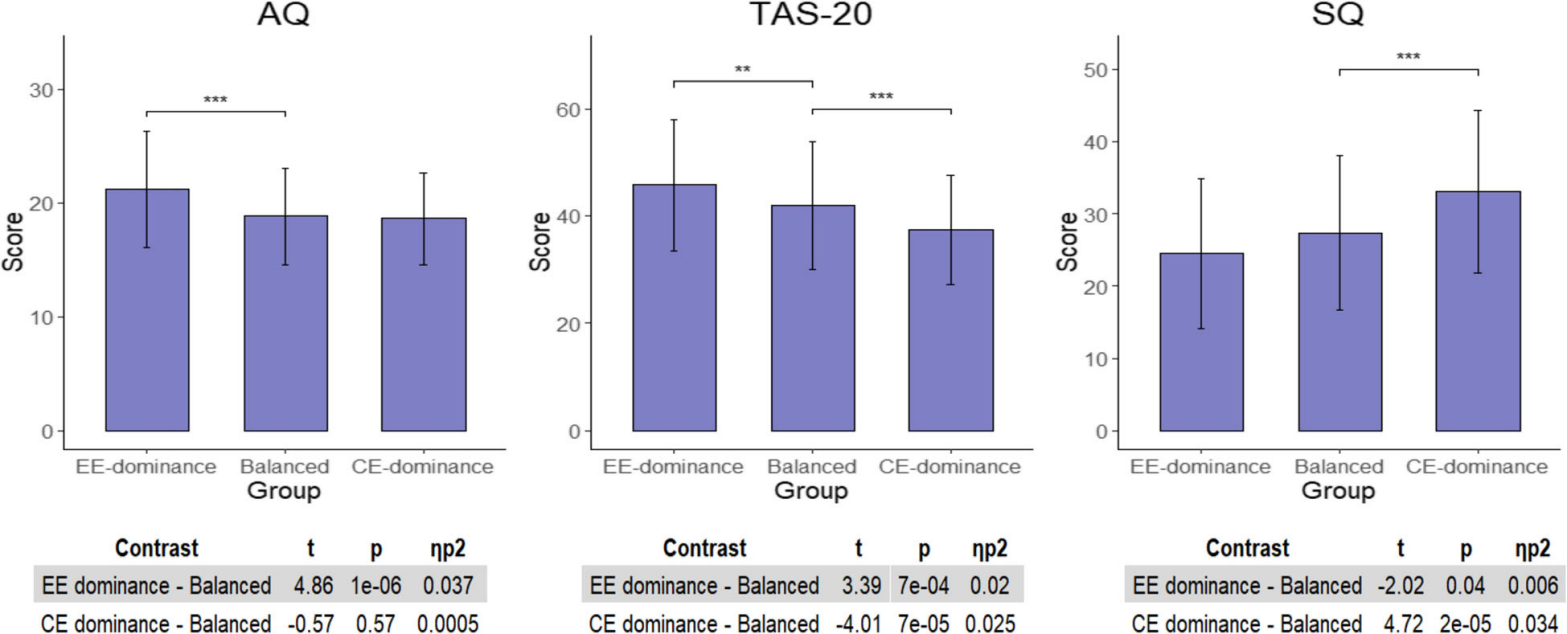
IRI-derived ED further analysis. Results of further analyses showing differences between high IRI-derived ED groups (EE-dominance and CE-dominance) and the balanced empathy group in AQ, TAS-20, and SQ. IRI, Interpersonal Reactivity Index; AQ, Autism Quotient; TAS-20, Toronto Alexithymia Scale; SQ, Systemizing Quotient. *p < 0.01, **p < 0.001, ***p < 0.0001

To better understand the origins of these associations, further analyses were conducted contrasting each of the ED groups (EE-dominance and CE-dominance, separately) with the balanced empathy group. To account for multiple testing, we used a strict Bonferroni-corrected *p* < 0.01 (0.05/3 significant measures × 2 contrasts). The results of these analyses are summarized in Fig. 2. AQ and TAS-20 subscales analyses are displayed in Supplementary Figure 1, Additional File 1. Analyzing the total AQ score revealed higher autism traits in individuals with EE-dominance (*t*(619) = 4.86, *p* = 1 × 10^−6^, *ƞ*_*p*_^2^ = 0.037). No difference was found between the CE-dominance and balanced empathy groups (*t*(619) = − 0.57, *p* = 0.57, *ƞ*_*p*_^2^ = 0.0005). Individuals with balanced empathy scored lower on TAS-20, as compared to the EE-dominance group (*t*(619) = 3.39, *p* = 7 × 10^−4^, *ƞ*_*p*_^2^ = 0.02) and scored higher compared to the CE-dominance group (*t*(619) = − 4.01, *p* = 7 × 10^−5^, *ƞ*_*p*_^2^ = 0.025). Our analyses also revealed significantly higher SQ score in the CE-dominance group as compared to the balanced empathy group (*t*(619) = 4.72, *p* = 2 × 10^−5^, *ƞ*_*p*_^2^ = 0.034). Nominally significant lower SQ scores were found in the EE-dominance group compared to the balanced empathy group (*t*(619) = − 2.02, *p* = 0.04, *ƞ*_*p*_^2^ = 0.006).

#### EQ-derived ED analyses

The same analyses were conducted calculating ED using the EQ. Similarly to the IRI analysis, multiple regression, with age and sex as covariates, showed ED-magnitude to be positively correlated with AQ score and uniquely contributed to its prediction (*β* = 0.12, *p* = 0.0045). In this analysis, the total EQ score also predicted AQ (*β* = − 0.3, *p* = 5 × 10^−12^). AQ score was neither explained by age (*β* = 0.06, *p* = 0.12) nor sex (*β* = 0.08, *p* = 0.075). Findings were similar when controlling for EE and CE (instead of a total empathy score), a positive correlation between ED-magnitude and AQ score (*β* = 0.13, *p* = 0.002). Neither age (*β* = 0.06, *p* = 0.14) nor sex (*β* = 0.04, *p* = 0.36) predicted AQ score. AQ score was also negatively correlated with CE (*β* = − 0.2, *p* = 2 × 10^−5^), but no correlation was found between AQ score and EE (*β* = − 0.07, *p* = 0.144).

After grouping the participants based on their EQ-derived ED score (see Table 3), no age differences were found between the three groups (*F* = 0.55, *p* = 0.57, *ƞ*_*p*_^2^ = 0.002). However, the total EQ score significantly differed between the groups (*F* = 3.141, *p* = 0.044, *ƞ*_*p*_^2^ = 0.01), so that the EQ score in the CE-dominant ED group was slightly lower than the EQ score in the balanced empathy group (*t*(668) = − 2.45, *p* = 0.015, *ƞ*_*p*_^2^ = 0.01). No difference in EQ score was found between the EE-dominance and balanced empathy groups (*t*(668) = − 1.02, *p* = 0.31, *ƞ*_*p*_^2^ = 0.001). Chi-square analyses revealed sex differences in both EE-dominance and CE-dominance groups (χ^2^(1, *N* = 109) = 8.33, *p* = 0.004, and χ^2^(1, *N* = 113) = 21.7, *p* < 0.0001, respectively) so that the EE-dominance group included more females than males (70%), and the CE-dominance ED group included more males than females (65%).

Results of the one-way ANOVA examination of autism-related traits between the three groups are described in Table 5 (AQ and TAS-20 subscales results are described in Supplementary Table 1 and Supplementary Table 2, respectively). These analyses revealed significant differences between the three groups in AQ and SQ scores. No differences in TAS-20 were found.

**Table 5:** EQ-derived ED analysis of empathy groups

	EE-dominance (***N*** = 110)		Balanced (***N*** = 442)		CE-dominance (***N*** = 119)		***p*** value	***F***	***η***_***p***_^**2**^
	Mean	SD	Mean	SD	Mean	SD			
AQ**	20.6	3.75	18.83	3.45	19.22	4.78	0.001	6.69	0.02
TAS-20	41.36	10.43	41.24	11.97	43.59	12.89	0.39	0.94	0.003
SQ**	23.62	10.29	27.98	10.88	31.7	11.05	0.0004	7.96	0.025

One-way ANOVA analyses results of the differences in AQ, TAS-20, and SQ scores between ED groups derived from EQ. *EQ* Empathy Quotient, *AQ* Autism-Spectrum Quotient, *TAS-20* Toronto Alexithymia Scale, *SQ* Systemizing Quotient

*p < 0.017, **p < 0.0017, ***p < 0.00017

**Fig. 3:**
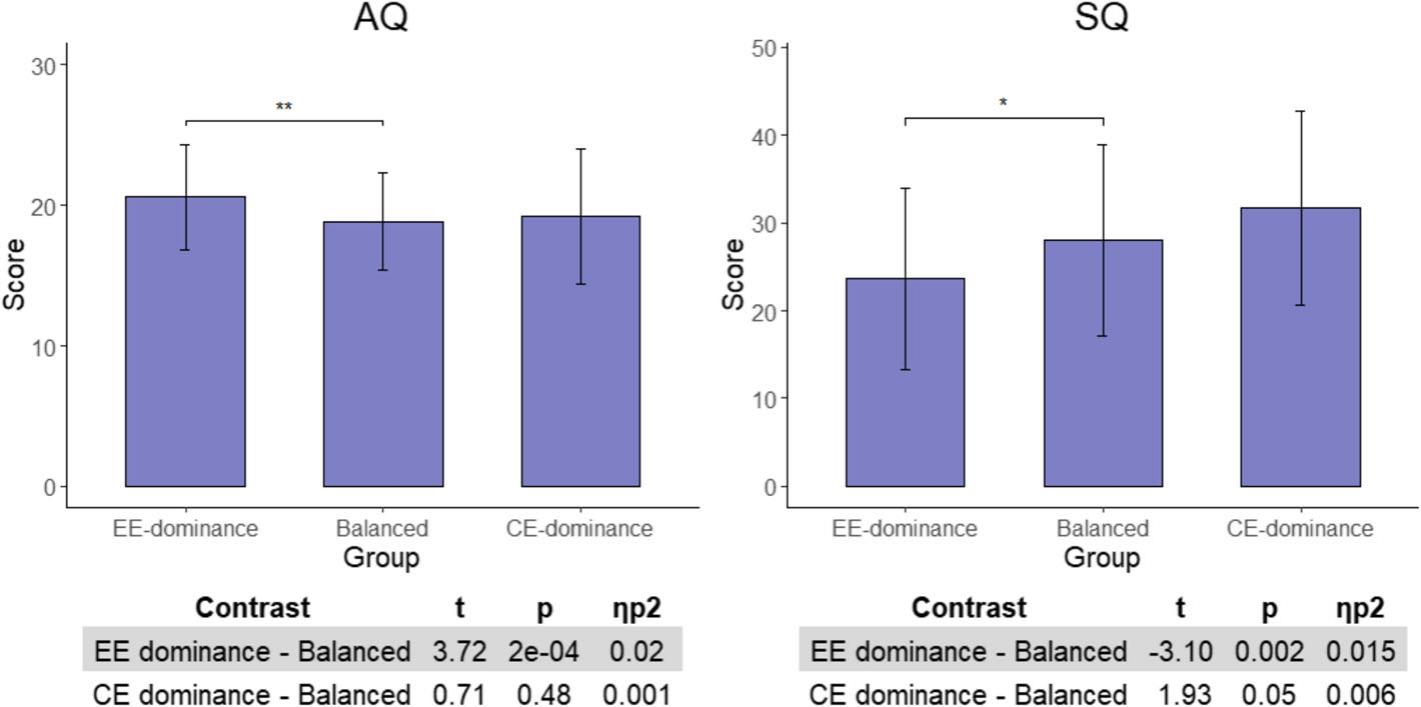
EQ-derived ED further analysis. Differences between ED (EE-dominance and CE-dominance) and balanced empathy groups derived from the EQ measure in AQ and SQ scores. AQ, Autism Quotient; SQ, Systemizing Quotient. *p < 0.0125, **p < 0.00125, ***p < 0.000125

Applying the same method used in the IRI analyses for the EQ-derived ED groups, further analyses were conducted contrasting the ED groups (CE-dominance and EE-dominance separately) with the balanced empathy group. Multiple testing was accounted for using a strict Bonferroni-corrected *p* < 0.0125 (0.05/2 significant measures × 2 contrasts). The results of this analysis are displayed in Fig. 3. Similarly to the IRI analyses, the contrasts examining the AQ scores between EE-dominance and balanced empathy groups revealed a significant difference, indicating higher autism traits in the EE-dominance group (*t*(623) = 3.72, *p* = 2 × 10^−4^, *ƞ*_*p*_^2^ = 0.02). No such differences were found between the balanced empathy and the CE-dominance group (*t*(619) = 0.71, *p* = 0.48, *ƞ*_*p*_^2^ = 0.001). Relative to individuals in the balanced empathy group, lower SQ score was found in the EE-dominance group (*t*(623) = − 3.1, *p* = 0.002, *ƞ*_*p*_^2^ = 0.015). SQ did not differ between the balanced empathy group and the CE-dominance group (*t*(619) = 1.93, *p* = 0.05, *ƞ*_*p*_^2^ = 0.006).

Analyses of the relationship between ED and the subscales of AQ and TAS-20 in addition to analyses examining ED as calculated based on the mean of EQ and IRI, as well as based on compatible groupings on both measures, can be found in Additional File 1.

## Discussion

In the current study, we investigated whether the intra-personal empathy imbalance between the cognitive and emotional components, a measure we termed ED, is associated with autism traits at the neurotypical range of the autism spectrum. Our analyses revealed that the size of ED is positively correlated with autism traits. We further showed that autism traits are elevated specifically in a group of individuals with relatively higher EE than CE. These results were robust and consistent across two different highly validated measures of empathy. ED groups also associated with other autism-related traits showing differences in systemizing and alexithymia.

The positive correlation between ED-magnitude and autism traits supports the novel notion that measuring the imbalance between the cognitive and emotional components of empathy can provide an informative and meaningful predictor of ASC-related traits in the general population. We suggest that the previous attempt by Cox et al. [50] to link autism traits to empathy imbalance in the general population fell short due to lack of power (small sample size of *N* = 18).

We next showed that ED association with autism traits could not be merely explained by participants’ general ability to empathize, as the later was controlled for in our regression analysis. This finding is also reflected in the ED group analyses, revealing no general empathy differences between EE-dominance and the balanced empathy groups in both measures of empathy. These results suggest that ED and the interplay between CE and EE are independent predictors of autism and autism-related traits.

Further investigating ED, we specifically discovered higher autism traits in individuals showing EE-dominant ED. This finding provides empirical evidence supporting the general notion of EIH which argues that the cognitive and behavioral characteristics of individuals with ASC are an adaptive response to over-arousal caused by the imbalance between CE and EE [36]. This suggests a novel hypothesis, to be tested in future research, that certain ASC group(s) can be characterized better through the concept of ED, rather than deficits in EE or CE separately. We further suggest that our results might address the conundrum of the mixed or heterogeneous findings in previous studies. For instance, it is possible that groups of individuals with autism who show unimpaired CE (e.g. [4]) also show higher than typical EE, and this could shed light on their experienced social difficulties. Therefore, individuals with autism who display typical levels of CE might be better understood through the lens of ED.

Further examining ED in autism might also shed light on the phenotypical heterogeneity of ASC that is apparent at the neural level [66]. For instance, fMRI studies reveal conflicting evidence of both increased and decreased resting-state functional connectivity [67, 68]. In their paper, Cox et al. [50] showed that dominance of EE was associated with stronger resting-state functional connectivity between socio-emotional regions of the brain such as the ventral anterior insula, orbital-frontal cortex, amygdala, and perigenual anterior cingulate. Connectivity between these regions was found to be altered in individuals with ASC [69, 70]. Furthermore, hyper-connectivity between the amygdala and the ventral anterior insula, regions which are co-activated by emotional stimuli [71], was previously associated with anxiety [72]. As might be hypothesized based on the EIH, individuals with EE-dominance might display elevated autism traits alongside over-arousal and anxiety elicited by sensitivity to external stimuli. Taken together, it is reasonable to hypothesize that this hyper-connectivity in socio-emotional networks might reflect a neural propensity defining a subgroup of individuals specifically affected by EE-dominance, and future studies could ascertain this hypothesis.

To better understand the features of the EE-dominant ED group, we examined associations with autism-related traits in the general population. We found that the EE-dominant group is characterized by intact or even lower systemizing propensity. This finding is surprising as this group was also related to higher AQ, while higher systemizing is characteristic of ASC [53, 73]. This seeming disparity might be explained by the specific association previously found between systemizing and the non-social aspects of autism (i.e., repetitive behavior and stereotyped interests). For instance, a recent genome-wide association study (GWAS) found genetic correlation between systemizing and the non-social, but not with the social aspects of ASC (i.e. social interaction and communication) [74]. Together with the lower systemizing found in the EE-dominant ED group, this might hint that EE-dominant ED is particularly related to the social difficulties of ASC, but not the non-social aspects of ASC.

The link between EE-dominant ED and social deficits prevalent in autism gains further support as individuals in this group showed heightened alexithymia in the IRI-derived ED. Alexithymia reflects difficulties in identifying and describing own emotions [51, 75], and although alexithymia is not a diagnostic feature of ASC it is widely prevalent in individuals with autism. It is also common in relatives of individuals with ASC and was suggested to be a feature of the broader autism phenotype [76]. As alexithymia and ASC share many overlapping features, alexithymia was suggested to play a complex role in ASC and to contribute to the social and emotional deficits displayed in autism [51, 77–79]. Nevertheless, the current finding should be taken cautiously as no differences in TAS-20 were found in the EQ-derived ED group analysis. Importantly, the empathy constructs tapped by the IRI and EQ are not identical, such that the IRI-derived EE subscale contains both empathic concern and personal distress, while the EQ-derived EE subscale focuses on one’s own emotional response to others’ emotions, and these differences might explain the inconsistent result. More specifically, ED as measured by the IRI, highlights a response to negative states/emotions, encompassing both empathic concern and personal distress as measures of EE. In contrast, the EQ-derived EE does not tap into personal distress caused by the other’s negative emotions. Rather it focuses on more neutral valenced states/emotions and more generally on the emotional reactions to others’ emotional states. Indeed, the replication of the main findings across these two different measures (and using different methods of calculating ED based on the two measures, see Additional File 1) and conceptualizations of EE strengthens the generalizability of our findings. At the same time, this distinction might explain our findings as alexithymia components, measured by the TAS-20, were previously associated with negative but not positive emotions [80, 81].

Finally, investigating the specific pattern of sex differentiation between ED groups in our data is also of interest. We found a significantly higher female-to-male ratio in the EE-dominant group, higher male-to-female ratio in the CE-dominant group, while no sex differences were found in the balanced empathy group. This finding is surprising as the EE-dominant group was associated with higher autism traits, which are far more prevalent in males than in females in clinical and in the general population [73, 82, 83]. This seeming discrepancy may in fact serve as a hint that ED might be particularly related to female-typical manifestations of autism, although this needs to be interpreted cautiously as females are over-represented in our sample and the opposite is true for autism as a diagnosis [84]. Furthermore, as females diagnosed with ASC is a relatively under-studied population that is not well characterized [84–86], we suggest that examination of sex differences in future research of ED in autism will be of value.

Interestingly, sex differentiation in empathy imbalance was previously suggested in the EIH, suggesting the theory that natural selection acted separately on EE and CE, shaping the interaction between the two capacities in a sex-dependent way [38]. According to this theory, men will be more susceptible than females to empathy imbalance and would facilitate male competitive, aggressive, and violent behavior. Conversely, reduced imbalance between the two capacities will characterize females and would facilitate behaviors such as child-rearing, tendencies selected for during human evolution [87]. The current findings show that the level of ED does not differentiate between the sexes. Rather, both males and females show ED but females tend to show EE-dominance whereas males tend to show CE-dominance. These results beg further investigation into the possible social and biological origins of this difference.

## Limitations

It is important to stress that all traits measured in this study were self-report questionnaires reflecting the participants’ perception of their own functioning and ability. Although all measures used are highly validated and were previously found to be correlated with other self-report and behavioral measures [14, 53, 56, 63, 88], self-report measures are prone to be over/underestimation of the subjects’ actual abilities. Thus, future research should address this limitation, using behavioral measures of empathy to further validate the ED concept.

Another limitation in the current study is the attempt to investigate autism traits solely within the neurotypical range, and not including participants diagnosed with ASC. On the one hand, we did not examine whether the population was truly neurotypical using diagnostic measures, although only five participants in the sample scored above the AQ clinical cut off [56]. On the other hand, examining autism traits in a sample of individuals seemingly at the neurotypical range limits the interpretability of the findings to the typical range of the autism spectrum. Future studies should examine these effects in a sample of individuals with ASC. Indeed, the effect sizes reported here are small to moderate. If a greater imbalance between EE and CE is associated with a diagnosis, stronger effect sizes can be expected.

The dissociation between EE and CE characterizes a wide range of psychiatric conditions [16, 39–41]; therefore, future studies should explore the role of ED in other clinical conditions. In this regard, it should also be mentioned that although we suggest ED is related to clinical conditions, the CE-dominant ED group was not characterized by higher autism traits nor alexithymia. However, it did show relatively higher systemizing scores and this finding should be further explored in other clinical traits associated with both higher systemizing and empathy, such as obsessive-compulsive personality disorder [89] and positive symptoms in schizophrenia [90]. Investigating other clinical traits with known empathy deficits such as anti-social personality disorder [16] should also be of interest.

## Conclusions

In this study, we showed that the intra-personal imbalance between the emotional and cognitive aspects of empathy offers a very novel way of understanding and measuring empathy as a construct and we predict is likely to be of prognostic value to autism traits in the general population towards early diagnosis and prevention. Our results are robust and were consistent using two different highly validated measures of empathy.

Our study adds to the understanding of the entire autism spectrum and sheds light on its mechanisms and variability in the general population. Based on empirical findings supporting empathic disequilibrium, the findings imply the possibility of novel subgroup classification of ASC based on the imbalance between EE and CE, rather by CE or EE separately, thus bridging the gap on current issues in the literature of ASC.

## Supplementary information

**Additional file 1. **Results and discussion of supplementary analyses. **Supplementary Table 1**. AQ subscales analyses. **Supplementary Table 2.** TAS-20 subscales analyses. **Supplementary Table 3.** Combined empathy score analyses. **Supplementary Table 4.** Concurrent group analyses. **Supplementary Figure 1**. Further analyses of the AQ and TAS-20 subscales based on IRI-derived ED groups. **Supplementary Figure 2**. Further analyses of the AQ subscales based on EQ-derived ED groups.

## Data Availability

The datasets generated and/or analyzed during the current study are not publicly available because consent from participants did not cover data sharing. The data sets are available from the corresponding author on reasonable request.

## Abbreviations

ASC: Autism spectrum conditions
EE: Emotional empathy
CE: Cognitive empathy
EIH: Empathy imbalance hypothesis
RMET: Reading the Mind in the Eyes Test
REA: Relative empathic ability
ED: Empathic disequilibrium
IRI: Interpersonal Reactivity Index
PT: Perspective taking
F: Fantasizing
EC: Empathic concern
PD: Personal distress
EQ: Empathy Quotient
AQ: Autism-Spectrum Quotient
TAS-20: Toronto Alexithymia Scale
SQ: Systemizing Quotient
