# Supplementary material for: Empathic disequilibrium in two different measures of empathy predicts autism traits in neurotypical population
Source: Mol Autism. 2020 Jul 13;11:59. doi: 10.1186/s13229-020-00362-1 (PMC7359469; doi:10.1186/s13229-020-00362-1)
Supplement: Supplementary file 1 — Additional file 1. Results and discussion of supplementary analyses. Supplementary Table 1. AQ subscales analyses. Supplementary Table 2. TAS-20 subscales analyses. Supplementary Table 3. Combined empathy score analyses. Supplementary Table 4. Concurrent group analyses. Supplementary Figure 1. Further analyses of the AQ and TAS-20 subscales based on IRI-derived ED groups. Supplementary Figure 2. Further analyses of the AQ subscales based on EQ-derived ED groups. [file 13229_2020_362_MOESM1_ESM.docx]

**Additional file 1**

In order to understand further the implications of Empathic Disequilibrium (ED) on the autism-related measures described in the main text, we conducted exploratory analyses of the subscales of the Autism-Spectrum quotient (AQ) [[1](#_ENREF_1)] and the Toronto Alexithymia Scale-20 (TAS-20) [[2](#_ENREF_2)]. Additionally, we conducted additional analyses to investigate ED groups based on two additional measures, one derived from IRI and EQ combined score, and the second based on a subsample of individuals who maintained their group assignment based on both measures.

In the below analyses we followed the same methodology described in the main article. Meaning, we calculated two separate ED measures derived from Interpersonal Reactivity Index (IRI) [[3](#_ENREF_3)] and/or from the Empathy Quotient (EQ) [[4](#_ENREF_4)]. We then grouped the participants according to their ED scores to cognitive empathy (CE)-dominant (**≥** 1 SD), emotional empathy (EE)-dominant (≤ -1 SD) and balanced empathy (between -1 to 1 SD) groups. We next examined the differences between the groups in each of the AQ and TAS-20 subscales.

**AQ Subscales Analyses**

For ED derived from IRI and EQ, the five AQ subscales were analyzed using one-way ANOVA contrasting each of the empathy groups (EE-dominance/CE-dominance/balanced empathy). Sex was used as a covariate. We assigned a strict Bonferroni-corrected p-value of (0.05 / 5 tests = 0.01) to account for multiple testing. Results are shown in Supplementary Table 1.

**Supplementary Table 1. AQ subscales analyses.**

| IRI |  |  |  |  |  |  |  |  |  |
| --- | --- | --- | --- | --- | --- | --- | --- | --- | --- |
|  | **EE-dominance (N=107)** | | **Balanced (N=409)** | | **CE-dominance (N=112)** | | **p-value** | **F** | **η_p_^2^** |
|  | **mean** | **SD** | **mean** | **SD** | **mean** | **SD** |  |  |  |
| Social skill | 3.75 | 1.5 | 3.36 | 1.37 | 3.3 | 1.38 | 0.05 | 3.1 | 0.01 |
| Attention switching*** | 4.8 | 2.13 | 3.94 | 2.06 | 3.66 | 2.15 | 2x10^-5^ | 11.05 | 0.035 |
| Attention to detail | 5.38 | 1.97 | 5.44 | 2 | 5.66 | 1.83 | 0.23 | 1.46 | 0.005 |
| Communication | 3.53 | 1.59 | 3.12 | 1.4 | 3.2 | 1.45 | 0.037 | 3.32 | 0.01 |
| Imagination*** | 3.7 | 1.72 | 2.98 | 1.5 | 2.8 | 1.37 | 7.5x10^-7^ | 14.42 | 0.045 |
|  |  |  |  |  |  |  |  |  |  |
| EQ |  |  |  |  |  |  |  |  |  |
|  | **EE-dominance (N=110)** | | **Balanced (N=442)** | | **CE-dominance (N=119)** | | **p-value** | **F** | **η_p_^2^** |
|  | **mean** | **SD** | **mean** | **SD** | **mean** | **SD** |  |  |  |
| Social skill*** | 4 | 1.47 | 3.39 | 1.37 | 2.94 | 1.26 | 6x10^-7^ | 14.66 | 0.045 |
| Attention switching | 4.48 | 1.84 | 3.98 | 2.16 | 3.85 | 2.16 | 0.01 | 4.27 | 0.014 |
| Attention to detail* | 5.22 | 1.93 | 5.43 | 1.96 | 5.84 | 1.99 | 0.005 | 5.31 | 0.017 |
| Communication*** | 3.98 | 1.41 | 3.04 | 1.38 | 3.05 | 1.51 | 8x10^-9^ | 19.16 | 0.06 |
| Imagination | 2.91 | 1.48 | 2.99 | 1.5 | 3.54 | 1.66 | 0.017 | 4.08 | 0.013 |
|  |  |  |  |  |  |  |  |  |  |

One-way ANOVA analyses results of the differences in AQ subscales scores between ED groups. ED derived from IRI is on the top of the table, while ED derived from EQ is on the bottom. IRI, Interpersonal Reactivity Index; EQ, Empathy Quotient; AQ, Autism Spectrum Quotient.
* *p* < 0.01, ** *p* < 0.001, *** *p* <0.0001.

**TAS-20 subscales analyses**

The same analyses were conducted on the three TAS-20 subscales. We assigned a Bonferroni-corrected p-value of (0.05/3 tests = 0.017) to account for multiple testing. Results are shown in Supplementary Table 2.

**Supplementary Table 2. TAS-20 subscales analyses.**

| IRI |  |  |  |  |  |  |  |  |  |
| --- | --- | --- | --- | --- | --- | --- | --- | --- | --- |
|  | **EE-dominance (N=107)** | | **Balanced (N=409)** | | **CE-dominance (N=112)** | | **p-value** | **F** | **ηp2** |
|  | **mean** | **SD** | **mean** | **SD** | **mean** | **SD** |  |  |  |
| Difficulty describing feelings*** | 12.55 | 4.78 | 11.2 | 4.5 | 9.77 | 3.84 | 3x10-6 | 13.11 | 0.04 |
| Difficulty identifying feelings** | 15.12 | 5.93 | 14.24 | 5.51 | 12.2 | 4.72 | 0.0003 | 8.32 | 0.026 |
| Externally-oriented thinking*** | 18.09 | 5.55 | 16.37 | 4.62 | 15.37 | 4.24 | 1x10-6 | 14.12 | 0.044 |
|  |  |  |  |  |  |  |  |  |  |
| EQ |  |  |  |  |  |  |  |  |  |
|  | **EE-dominance (N=103)** | | **Balanced (N=415)** | | **CE-dominance (N=108)** | | **p-value** | **F** | **ηp2** |
|  | **mean** | **SD** | **mean** | **SD** | **mean** | **SD** |  |  |  |
| Difficulty describing feelings | 11.15 | 4.29 | 10.93 | 4.48 | 12.02 | 4.78 | 0.2 | 1.66 | 0.005 |
| Difficulty identifying feelings | 14.18 | 5.04 | 14 | 5.59 | 13.98 | 5.71 | 0.96 | 0.04 | 0.0001 |
| Externally-oriented thinking | 16.02 | 4.15 | 16.31 | 4.83 | 17.59 | 5.07 | 0.25 | 1.4 | 0.004 |

One-way ANOVA analyses results of the differences in TAS-20 subscales scores between ED groups. ED derived from IRI is on the top of the table, while ED derived from EQ is on the bottom. IRI, Interpersonal Reactivity Index; EQ, Empathy Quotient; TAS-20, Toronto Alexithymia Scale.

* *p* < 0.017, ** *p* < 0.0017, *** *p* <0.00017.

***Further analysis of AQ and TAS-20 subscales***

*Analyses using the IRI derived ED score.* Consistent with the analyses in the main article, further analyses were conducted contrasting the ED groups (EE-dominance and CE-dominance, separately) with the balanced empathy group for the two significant AQ subscales (‘attention switching’ and ‘imagination’) and the three significant TAS-20 subscales (‘difficulty describing feelings’, ‘difficulty identifying feelings’, and ‘externally-oriented thinking’). To account for multiple testing, we used a strict Bonferroni corrected p < 0.01 (0.05 / 5 significant measures x 2 contrasts). Results of the analysis are presented in Supplementary Figure 1.

**Supplementary Figure 1. Further analyses of the AQ and TAS-20 subscales based on IRI-derived ED groups.**


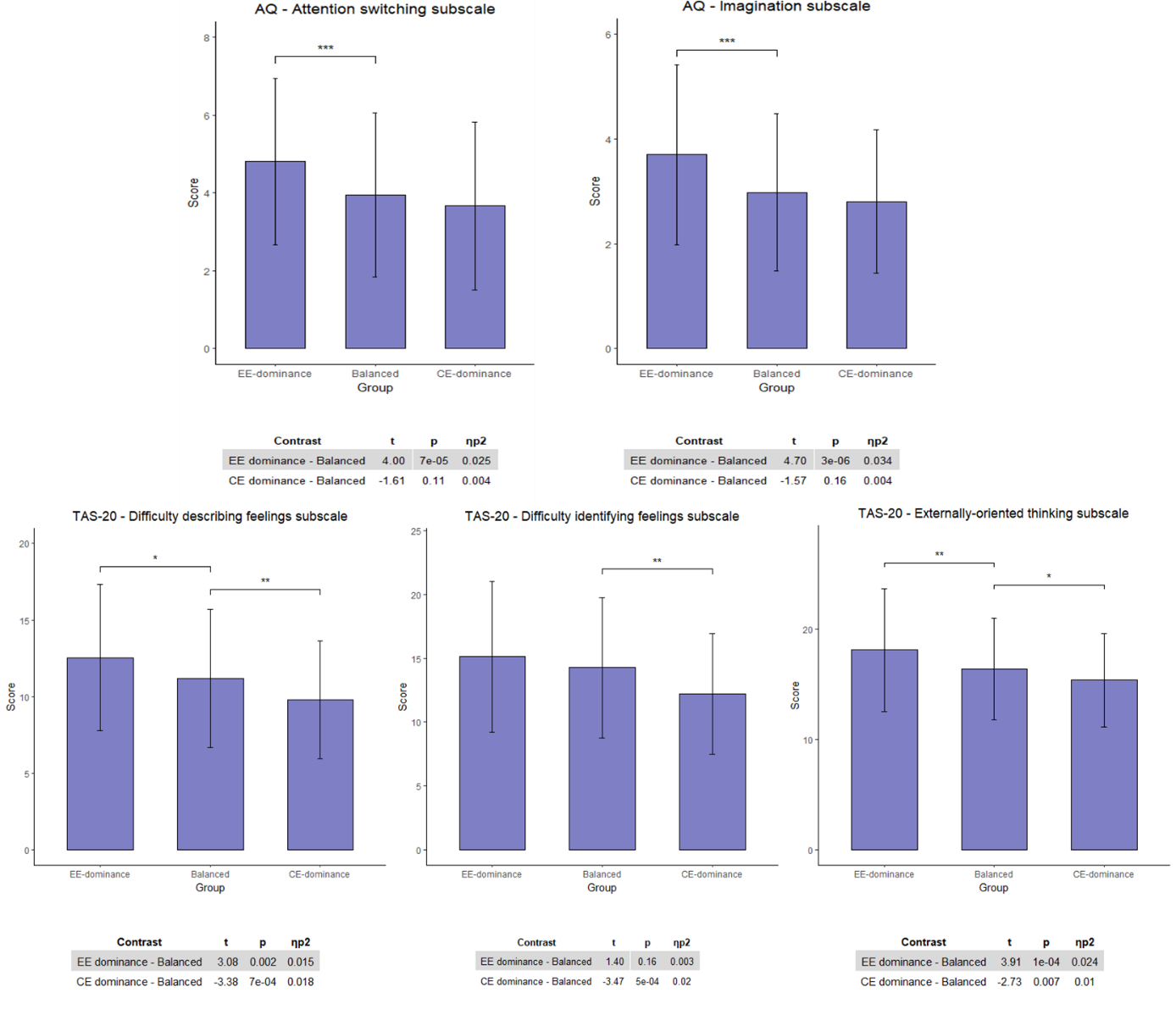


Results of further analyses showing differences between ED groups (EE dominance and CE dominance) and the balanced empathy group derived from the IRI measure in the AQ ‘attention switching’ and ‘imagination’ subscales (top panel), and all of the TAS-20 subscales (bottom panel). IRI, Interpersonal Reactivity Index; AQ, Autism Quotient; TAS-20, Toronto Alexithymia Scale.

* *p* < 0.01, ** *p* < 0.001, *** *p* < 0.0001.

*Analyses using the EQ derived ED score.* Consistent with the analyses in the main article, further analyses were conducted contrasting the ED groups (EE-dominance and CE-dominance, separately) with the balanced empathy group for the three significant AQ subscales (‘social skill’, ‘attention to detail’ and ‘communication’). To account for multiple testing, we used a strict Bonferroni corrected p < 0.0083 (0.05 / 3 significant measures x 2 contrasts). Results of the further analysis in the AQ subscales are presented in Supplementary Figure 2.

**Supplementary Figure 2. Further analyses of the AQ subscales based on EQ-derived ED groups.**


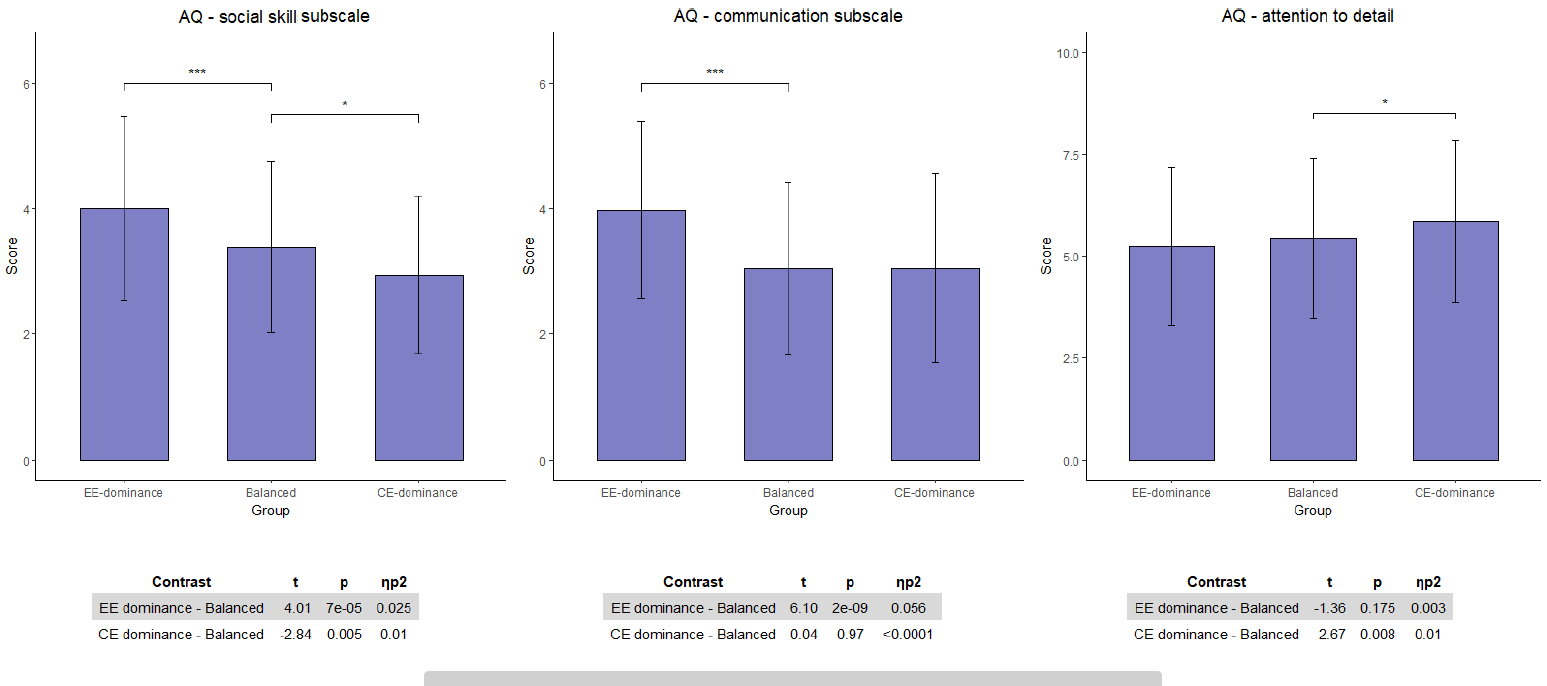


Results of further analyses showing differences between ED groups (EE dominance and CE dominance) and the balanced empathy group derived from the EQ in the AQ ‘social skill’, ‘attention to detail’, and ‘communication’ subscales. EQ, Empathy Quotient; AQ, Autism Quotient.

* *p* < 0.0083, ** *p* < 0.00083, *** *p* < 0.000083.

**Combined empathy score analyses**

We created a combined empathy score, calculated as the mean of EQ and IRI subscales scores. From this combined score we calculated ED, as previously described.

*Combined Group Demographics.* We next grouped participants based on their ED scores. Consistent with the results reported in the main text, participants in each group did not differ in age (*F* = 0.05, *p* = 0.95, *ƞ_p_^2^* = 0.0001) and total empathy score (*F* = 0.49, *p* = 0.61, *ƞ_p_^2^* = 0.002). Chi-square analyses revealed sex differences in both EE-dominance and CE-dominance groups (ꭓ^2^(1, *N* = 67) = 7.98, *p* = 0.0047 and ꭓ^2^(1, *N* = 74) = 16.68, *p* = 4x10^-5^, respectively) so that the EE-dominance group included more females than males (73%), and the CE-dominance ED group included more males than females (67.5%). No such differences were found in the balanced empathy group (ꭓ^2^(1, *N* = 487) = 0.15, *p* = 0.7).

*Group Analysis.* We assigned a Bonferroni-corrected p-value of (0.05/3 tests = 0.017) to account for multple testing. Results of the one-way ANOVA examination of autism-related traits between the three groups are described in Supplementary Table 3. These analyses revealed significant differences between the three groups in AQ, TAS-20 and SQ scores.

**Supplementary Table 3. Combined empathy score analyses.**

| Combined empathy score | | | | |  |  |  |  |  |  |  |
| --- | --- | --- | --- | --- | --- | --- | --- | --- | --- | --- | --- |
|  | **EE-dominance (N=67)** | | **Balanced (N=483)** | | **CE-dominance (N=74)** | | **p-value** | | **F** | **η_p_^2^** | |
|  | **mean** | **SD** | **mean** | **SD** | **mean** | **SD** |  | |  |  | |
| AQ*** | 21.52 | 5.00 | 18.95 | 4.34 | 18.7 | 4.00 | 3x10^-5^ | | 10.66 | 0.03 | |
| TAS-20** | 45.02 | 13.41 | 41.71 | 11.69 | 38.65 | 11.34 | 0.0007 | | 7.39 | 0.023 | |
| SQ*** | 21.34 | 9.05 | 27.99 | 10.96 | 33.22 | 10.41 | 8x10^-7^ | | 14.36 | 0.044 | |

One-way ANOVA analyses results of the differences in AQ, TAS-20, and SQ scores between ED groups derived from the combined empathy score (mean score IRI and EQ). AQ, Autism-Spectrum Quotient; TAS-20, Toronto Alexithymia Scale; SQ, Systemizing Quotient.

* *p* < 0.017, ** *p* < 0.0017, *** *p* <0.00017.

*Further analyses.* Consistent with the analyses in the main article, further analyses were conducted contrasting the ED groups (EE-dominance and CE-dominance, separately) with the balanced empathy group. To account for multiple testing, we used a strict Bonferroni corrected p < 0.01 (0.05 / 3 significant measures x 2 contrasts). Analysing the total AQ score revealed higher autism traits in individuals with EE-dominance (*t*(620) = 4.48, *p* = 9x10^-6^, *ƞ_p_^2^* = 0.031). No difference was found between the CE-dominance and balanced empathy groups (*t*(620) = 0.62, *p* = 0.54, *ƞ_p_^2^* = 0.0006). Individuals with balanced empathy scored nominally lower (as the corrected p-value is 0.01) on TAS-20, as compared to the EE-dominance group (*t*(620) = 2.54, *p* = 0.01, *ƞ_p_^2^* = 0.01) and scored nominally higher compared to the CE-dominance group (*t*(1, 619) = -2.59, *p* = 0.01, *ƞ_p_^2^* = 0.01). Our analyses also revealed significant lower SQ score in the EE-dominance group as compared to the balanced empathy group (*t*(620) = 4.22, *p* = 3x10^-5^, *ƞ_p_^2^* = 0.028) and higher SQ scores in the CE-dominance group compared to the balanced empathy group (*t*(620) = -2.83, *p* = 0.005, *ƞ_p_^2^* = 0.013).

**Compatible IRI and EQ analyses**

We also conducted another analysis based only on participants who were consistently classified in the same empathy group based on both IRI and EQ ED score. For example, only participants who were classified in the EE-dominant ED group in both the IRI and EQ were included in this analysis.

*Group Demographics.* Such analysis, as expected, reduced our power leaving a smaller sample of only 328 participants. Participants in each group did not differ in age (*F* = 0.055, *p* = 0.95, *ƞ_p_^2^* = 0.0004) and EQ scores (*F* = 0.84, *p* = 0.43, *ƞ_p_^2^* = 0.005). However, they did differ in IRI scores (*F* = 9.86, *p* = 6x10^-5^, *ƞ_p_^2^* = 0.057). Specifically, EE-dominant ED group showed higher IRI scores than the balanced group (*t*(325) = 2.2, *p* = 0.03, *ƞ_p_^2^* = 0.015), while CE-dominant ED group scored lower on the IRI (*t*(325) = 3.66 , *p* = 0.0003, *ƞ_p_^2^* = 0.04). To control for these differences, we used ANCOVA in the group analysis, controlling for IRI scores. Chi-square analyses revealed sex differences in both CE-dominance and EE-dominance groups (ꭓ^2^(1, *N* = 28) = 10.92, *p* = 0.001 and ꭓ^2^(1, *N* = 21) = 5.31, *p* = 0.02, respectively) so that the EE-dominance group included more females than males (81%), and the CE-dominance group included more males than females (75%). No such differences were found in the balanced empathy group (ꭓ^2^(1, *N* = 279) = 0.66, *p* = 0.41).

*Group Analysis.* We assigned a Bonferroni-corrected p-value of (0.05/3 tests = 0.017) to account for multiple testing. Results of the one-way ANCOVA examination of autism-related traits between the three groups are described Supplementary Table 4. These analyses revealed significant differences between the three groups in AQ and SQ scores.

**Supplementary Table 4.** **Concurrent group analyses.**

| Concurrent group analysis | | | | |  |  |  |  |  |  |  |
| --- | --- | --- | --- | --- | --- | --- | --- | --- | --- | --- | --- |
|  | **EE-dominance (N=21)** | | **Balanced (N=279)** | | **CE-dominance (N=28)** | | **p-value** | | **F** | **η_p_^2^** | |
|  | **mean** | **SD** | **mean** | **SD** | **mean** | **SD** |  | |  |  | |
| AQ*** | 21.9 | 4.18 | 18.38 | 4.23 | 17.39 | 3.06 | 0.00016 | | 8.97 | 0.053 | |
| TAS-20 | 41.81 | 9.28 | 41.05 | 11.85 | 38.36 | 9.28 | 0.04 | | 3.17 | 0.02 | |
| SQ** | 20.33 | 9.08 | 27.54 | 10.57 | 35.89 | 9.43 | 0.0003 | | 8.32 | 0.05 | |

One-way ANCOVA analyses results of the differences in AQ, TAS-20, and SQ scores between ED groups as created by consistent classification be ED scores derived from both IRI and EQ. AQ, Autism-Spectrum Quotient; TAS-20, Toronto Alexithymia Scale; SQ, Systemizing Quotient.

* *p* < 0.017, ** *p* < 0.0017, *** *p* <0.00017.

*Further analyses.* Further analyses were conducted contrasting the ED groups (EE-dominance and CE-dominance, separately) with the balanced empathy group. To account for multiple testing, we used a strict Bonferroni corrected p < 0.0125 (0.05 / 2 significant measures x 2 contrasts). Analysing the total AQ score revealed higher autism traits in individuals with EE-dominance (*t*(325) = 3.91, *p* = 0.0001, *ƞ_p_^2^* = 0.045). No difference was found between the CE-dominance and balanced empathy groups (*t*(325) = 1.47, *p* = 0.14, *ƞ_p_^2^* = 0.007). Our analyses also revealed significant lower SQ score in the EE-dominance group as compared to the balanced empathy group (*t*(325) = 2.54, *p* = 0.012, *ƞ_p_^2^* = 0.02) and higher SQ scores in the CE-dominance group compared to the balanced empathy group (*t*(325) = -3.08, *p* = 0.002, *ƞ_p_^2^* = 0.028).

**Discussion**

Our supplementary analyses revealed that AQ scores were systematically higher in the EE-dominant ED group in both IRI and EQ analyses. However, differences in AQ subscales were found between the two measures. The IRI-derived ED score was associated with differences in the ‘attention switching’ and ‘imagination’ subscales, while the EQ-derived ED score was associated with differences in the ‘communication’ and ‘social skill’ subscales. Differences between the CE-dominant ED group and the balanced empathy group were found in the EQ-derived analysis revealing a lower AQ score in the ‘social skill’ subscale, and a higher AQ score in the ‘attention to detail’ subscale. Furthermore, alexithymia differences between groups were evidenced only in the IRI-derived groups, showing elevated scores in the TAS-20 ‘difficulties describing feelings’ and ‘externally-oriented thinking’ subscales in the EE-dominant ED group relative to the balanced empathy group. Lower alexithymia scores, relative to the balanced empathy group in all three TAS-20 subscales were evidenced in the CE-dominant ED group.

Although differences exist between IRI and EQ, the results from the combined and compatible score of ED replicated the results reported in the main text. This supports the validity of our findings and emphasizes ED as a meaningful construct, which is consistently associated with autism traits in neurotypical population, with little dependence on a specific measure.

Overall, EE-dominant ED group was found to be characterized by higher autism traits as reflected in four of the AQ subscales. The elevated AQ score in the EE-dominant ED is consistent with the general notion of empathy imbalance hypothesis (as discussed thoroughly in the main article) which argues that the cognitive and behavioral characteristics of individuals with Autism Spectrum Conditions (ASC) is an adaptive response to over-arousal caused by the imbalance between CE and EE [[5](#_ENREF_5)].

‘Attention to detail’ was the only AQ subscale that was found to be intact in the EE-dominant ED group in our analyses. It was previously suggested that ‘attention to detail’ is qualitatively different from the other AQ subscales as it emphasizes a non-social factor of ASC [[6](#_ENREF_6)]. This finding is consistent with the intact systemizing ability found in the EE-dominant ED group (as reported in the main article), and supports our suggestion that EE-dominant ED is specifically associated with the social difficulties of ASC, but not with the non-social aspects of ASC.

On the contrary, the ‘attention to detail’ subscale was found to be elevated in the CE-dominant ED group as measured only by the EQ-derived ED. This subscale represents a cognitive style in which one attends to fine-grained details of the world at the expense of integrative perception [[1](#_ENREF_1), [7](#_ENREF_7)]. Although this kind of cognitive organization is prevalent in individuals with ASC [[8](#_ENREF_8)], it is typically a non-social characteristic and is non-specific to ASC as it is common in other clinical conditions such as obsessive compulsive personality disorder [[9](#_ENREF_9)] and schizotypal personality disorder [[10](#_ENREF_10)]. This is consistent with our suggestion mentioned in the main article that the meaning of CE-dominant ED, and its relevance to other clinical traits should be explored in future studies. Additionally, the CE-dominant ED group was characterized by lower alexithymia scores and lower scores on the 'social skill' subscale of the AQ, which suggests a linear association between these traits, as compared to the other measures which predominantly showed associations with EE-dominant ED.

Of interest, a noticeable disparity emerges between the variability of autism traits that is captured by the EQ-derived ED as compared to the IRI-derived ED. We suggest that each ED measure captures slightly different aspects of the ED construct as reflected in differences between the two empathy measures. Primarily, EQ as well as its ED derivative, highlight a more social aspect of empathy (e.g. “I can easily tell if someone else wants to enter a conversation”). This might explain the association with the AQ ‘social skill’ and ‘communication’ subscales, which solely differed between groups using the EQ-derived ED. Alternatively, the IRI-derived ED contains the IRI ‘fantasizing’ subscale which emphasizes participants’ ability to imaginatively engage themselves in the feelings and actions of others [[3](#_ENREF_3)], thus explaining why IRI-derived ED is more sensitive to the ‘imagination’ subscale of AQ.

Another disparity between the ED measures is apparent in the alexithymia analysis. Higher TAS-20 scores in ‘difficulty describing feelings’ and the ‘externally-oriented thinking’ subscales were found in the EE-dominant ED group only in the IRI analysis but not in the EQ analysis. These results show that individuals with EE-dominant ED, find it difficult to verbally express and communicate their own feelings to others and are characterized by an externally oriented cognitive style. As suggested in the main article, EE, as measured by the IRI, contains a measure of the tendency to experience personal distress caused by the other’s negative emotions, while the EQ-derived ED focuses on a more neutral valence of ED. This disparity between the two measures might explain why alexithymia differences, as well as differences in the subscale, were found only in the IRI analysis, as TAS-20 subscales were previously associated with negative but not positive emotions [[11](#_ENREF_11), [12](#_ENREF_12)].

Taken together, these results highlight the robustness of our findings and suggest that although EQ and IRI have concurrent validity [[13](#_ENREF_13)], noticeable differences are apparent between the two ED measures derived from each questionnaire. This helps clarify the relatively undiscussed differences between the two measures, which is of importance in and of itself.

**References**

1. Baron-Cohen S, Wheelwright S, Skinner R, Martin J, Clubley E. The Autism-Spectrum Quotient (AQ): Evidence from Asperger Syndrome/High-Functioning Autism, Males and Females, Scientists and Mathematicians. Journal of Autism and Developmental Disorders. 2001;31(1):5-17.

2. Bagby RM, Parker JDA, Taylor GJ. The twenty-item Toronto Alexithymia scale—I. Item selection and cross-validation of the factor structure. Journal of Psychosomatic Research. 1994;38(1):23-32.

3. Davis MH. A multidimensional approach to individual difference in empathy. JSAS Catalog of Selected Documents in Psychology. 1980;10:85.

4. Baron-Cohen S, Wheelwright S. The Empathy Quotient: An Investigation of Adults with Asperger Syndrome or High Functioning Autism, and Normal Sex Differences. Journal of Autism and Developmental Disorders. 2004;34(2):163-75.

5. Smith A. The Empathy Imbalance Hypothesis of Autism: A Theoretical Approach to Cognitive and Emotional Empathy in Autistic Development. The Psychological Record. 2009;59(3):489-510.

6. Kitazoe N, Fujita N, Izumoto Y, Terada S-i, Hatakenaka Y. Whether the Autism Spectrum Quotient consists of two different subgroups? Cluster analysis of the Autism Spectrum Quotient in general population. Autism. 2016;21(3):323-32.

7. Stevenson RA, Toulmin JK, Youm A, Besney RMA, Schulz SE, Barense MD, et al. Increases in the autistic trait of attention to detail are associated with decreased multisensory temporal adaptation. Scientific Reports. 2017;7(1):14354.

8. Happé F, Frith U. The Weak Coherence Account: Detail-focused Cognitive Style in Autism Spectrum Disorders. Journal of Autism and Developmental Disorders. 2006;36(1):5-25.

9. Gadelkarim W, Shahper S, Reid J, Wikramanayake M, Kaur S, Kolli S, et al. Overlap of obsessive–compulsive personality disorder and autism spectrum disorder traits among OCD outpatients: an exploratory study. International Journal of Psychiatry in Clinical Practice. 2019;23(4):297-306.

10. Ford TC, Crewther DP. Factor Analysis Demonstrates a Common Schizoidal Phenotype within Autistic and Schizotypal Tendency: Implications for Neuroscientific Studies. Frontiers in psychiatry. 2014;5:117.

11. Suslow T, Donges U-S. Alexithymia Components Are Differentially Related to Explicit Negative Affect But Not Associated with Explicit Positive Affect or Implicit Affectivity. Frontiers in psychology. 2017;8:1758.

12. Zhang L, Ye R, Yu F, Cao Z, Zhu C, Cai Z, et al. How does emotional context modulate response inhibition in alexithymia: electrophysiological evidence from an ERP study. PLOS ONE. 2012;7(12):e51110-e.

13. Lawrence EJ, Shaw P, Baker D, Baron-Cohen S, David AS. Measuring empathy: reliability and validity of the Empathy Quotient. Psychological Medicine. 2004;34(5):911-20.
